# Higher femorotibial width ratio is associated with knee injury and osteoarthritis: A UK Biobank study of 61,795 participants

**DOI:** 10.1002/jeo2.70986

**Published:** 2026-11-13

**Authors:** Bilal Rayes, Mijin Jung, Fiona Saunders, Raja Ebsim, Asad Hashmi, Richard M. Aspden, Jenny Gregory, Timothy Cootes, Claudia Lindner, Simon G. F. Abram, Jonathan H. Tobias, Martyn Snow, Benjamin G. Faber

**Affiliations:** ^1^ Musculoskeletal Research Unit University of Bristol Bristol UK; ^2^ MRC Integrative Epidemiology Unit University of Bristol Bristol UK; ^3^ Aberdeen Centre for Research Excellence in Musculoskeletal Health University of Aberdeen Aberdeen UK; ^4^ Division of Informatics, Imaging and Data Science The University of Manchester Manchester UK; ^5^ Centre for Science and Technology in Medicine Keele University Staffordshire UK; ^6^ The Robert Jones and Agnes Hunt Hospital Oswestry, Shropshire UK

**Keywords:** epidemiology, imaging, knee injury, osteoarthritis, risk factor

## Abstract

**Purpose:**

Soft tissue knee injury and knee osteoarthritis (KOA) are major contributors to long‐term disability. Structural differences between femoral and tibial widths have been proposed as a risk factor, but population‐scale evidence is limited. This study aimed to examine the associations between femorotibial width ratio (FTWR) and knee injury and KOA in a large imaging cohort.

**Methods:**

FTWR was derived for the whole knee and both medial and lateral compartments using a machine‐learning pipeline applied to high‐resolution knee dual‐energy X‐ray absorptiometry scans from UK Biobank. Outcomes were soft tissue knee injury, ligament injury, meniscus injury, hospital‐diagnosed KOA, total knee replacement (TKR) and knee pain, identified using ICD (International Classification of Diseases) and OPCS‐4 (Office of Population Censuses and Surveys Classification of Interventions and Procedures, Version 4) codes from Hospital Episode Statistics (HES) and questionnaires. Clustered logistic regression estimated associations. Odds ratios (ORs) are reported per standard deviation increase in FTWR, adjusted for age, sex, height, weight and ethnicity.

**Results:**

Higher whole‐knee FTWR was associated with increased odds of soft tissue knee injury (OR 1.09, 95% confidence interval [CI] 1.06–1.13), ligament injury (1.07, 1.01–1.13) and meniscus injury (1.10, 1.05–1.14) in 61,795 participants. Associations were stronger for KOA outcomes, including hospital‐diagnosed KOA (1.16, 1.12–1.21), TKR (1.27, 1.17–1.39) and chronic knee pain (1.08, 1.05–1.10). Medial compartment FTWR mirrored these positive associations, while lateral FTWR showed weaker inverse associations.

**Conclusions:**

Femorotibial width discrepancy is consistently associated with knee injury and KOA across clinical endpoints. Future work is justified to see if FTWR can serve as a scalable imaging marker of knee injury risk and provide opportunities for targeted prevention.

**Level of Evidence:**

Level III.

## Graphical abstract

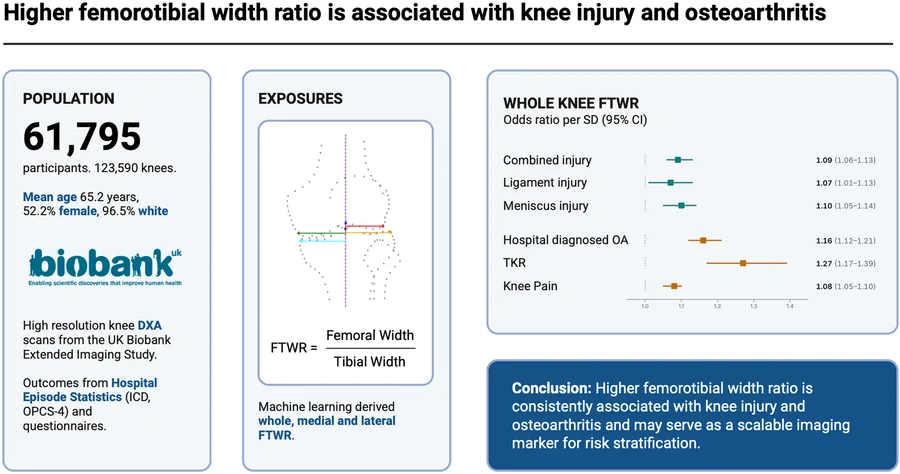

**Abbreviations**
ACL: anterior cruciate ligament
CI: confidence interval
DXA: dual‐energy X‐ray absorptiometry
FTWR: femorotibial width ratio
HES: Hospital Episode Statistics
HES‐KOA: hospital‐diagnosed knee osteoarthritis
ICD: International Classification of Diseases
KOA: knee osteoarthritis
LFCR: lateral femoral condyle ratio
OPCS: Office of Population Censuses and Surveys
OR: odds ratio
TFR: tibia:femur ratio
TKR: total knee replacement

## INTRODUCTION

Soft tissue knee injury and knee osteoarthritis (KOA) are major causes of musculoskeletal pain and disability [37]. In the United Kingdom, the demand for total knee replacement (TKR) is projected to rise by almost 40% in the coming decades [30]. Soft tissue injuries, including ligament and meniscus tears, are common in sporting populations and are well‐established risk factors for the subsequent development of KOA [15, 47, 50]. However, progression from knee injury to symptomatic KOA is variable, suggesting that underlying structural and biomechanical factors may influence long‐term disease progression [7].

Variations in the structural characteristics of knee joints can influence load distribution across femorotibial compartments and may therefore play an important role in the risk of acute injury or long‐term degenerative changes [2, 9, 19, 39]. Statistical shape modelling approaches have shown that global measures of knee shape derived from dual‐energy X‐ray absorptiometry (DXA) images are associated with KOA progression, independent of conventional radiographic features [3, 5]. While these methods provide valuable insights, they capture complex, multivariable aspects of joint morphology that are harder to interpret mechanistically.

Previous radiographic studies have shown that variation in femoral notch width or increased posterior tibial slope can increase the risk of anterior cruciate ligament (ACL) injury [17, 33, 41]. Similarly, lateral femoral condyle ratio (LFCR) [27] is also associated with an increased risk of ACL injury; however, radiographic analysis of these factors can be challenging. A simpler structural feature is the relative width of the femur and tibia at the knee joint. Discrepancy between femoral and tibial widths may influence joint stability and compartment‐specific load distribution during weight‐bearing exercise and dynamic activity [21, 22]. This could plausibly increase stress on articular cartilage and soft tissue structures, predisposing individuals to knee injury and osteoarthritis. Although many elements of knee morphology and alignment have previously been examined in relation to the risk of osteoarthritis, these studies have been limited by relatively small sample sizes [35, 38]. As a result, population‐level evidence on the associations of femorotibial width discrepancy with knee injury and osteoarthritis remains limited.

Advancements in large‐scale imaging datasets such as the UK Biobank now enable detailed assessment of joint morphology at unprecedented scale [29]. Using machine learning, it is now possible to derive compartment‐specific measures of femoral and tibial width from knee DXA images. Although DXA is primarily used for the assessment of bone mineral density, improvements in image resolution now permit reliable visualization and measurement of bony structures at the knee [51]. The femorotibial width ratio (FTWR), defined as the femoral width divided by the tibial width, provides a simple and interpretable measure of knee morphology. FTWR can be quantified across the whole joint and for medial and lateral compartments respectively, allowing the investigation of compartment‐specific associations with injury and osteoarthritis. This study aimed to (i) derive FTWR at population scale in the UK Biobank, (ii) investigate its associations with clinical outcomes relating to knee injury and (iii) investigate its associations with knee pain and osteoarthritis. It was hypothesized that FTWR is associated with knee injury, KOA, TKR and knee pain.

## METHODS

### Population

The UK Biobank is a longitudinal, prospective cohort study that recruited approximately 500,000 participants, aged 40–69 at recruitment, in the United Kingdom between 2006 and 2010 [29]. Health and lifestyle data were also collected, including questionnaires and biological samples. This study involved participants from the UK Biobank Extended Imaging Study, which began in 2014 and collected large‐scale imaging data, including high‐resolution knee DXA [6]. Only participants with images available for download on 1 January 2025 were included in this study.

### DXA‐derived measures of knee morphology

High‐resolution knee DXA scans were captured using a Lunar iDXA scanner (GE Healthcare) following a standardized protocol with the participants lying supine, minimizing positional variation [29]. A Random Forest machine learning algorithm (BoneFinder, University of Manchester [28]) was used to place 129 landmark points on the distal femur and proximal tibia. These points were placed inside osteophytes, if present, and therefore do not encompass them. The anatomical positions were automatically determined using the pre‐defined BoneFinder algorithm, which was trained using 7000 manually marked and checked left knee DXA images; details and validation of this method have been published previously [3, 5]. Right knee images were mirrored prior to landmarking and processing. Once outlined, a custom Python script was developed to identify femoral and tibial midpoints and calculate the total, medial and lateral femoral and tibial widths [11]. Femoral and tibial midpoints corresponded to points 22 and 65, respectively; lateral and medial femoral points were 16 and 28, and lateral and medial tibial points were 57 and 73. Distances between relevant landmark pairs were used to derive geometric compartment‐specific widths (Figure 1). The DXA images used in this study have a pixel dimension of approximately 0.25 × 0.25 millimetres (mm). The Digital Imaging and Communications in Medicine (DICOM) file format provides an image‐specific pixel size in millimetres. The Python code obtains the number of pixels between two landmark points, which can then be converted into millimetres for each image. FTWR was subsequently calculated by dividing femoral width by tibial width across the whole joint, medial and lateral compartments.

**Figure 1:**
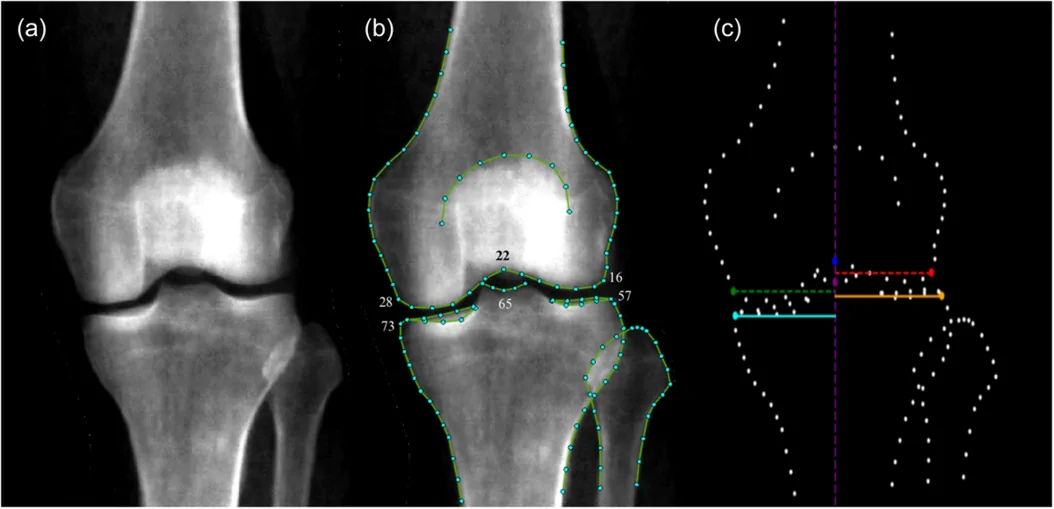
(a) High‐resolution knee DXA scan from UK Biobank. Reproduced with the kind permission of UK Biobank ©. (b) Outline points marked by a Random Forest machine learning algorithm. Selected anatomical landmarks used for width measurements are highlighted. (c) Custom Python script‐derived measures are shown in the right panel. Femoral and tibial midlines are shown as vertical dashed lines. Horizontal segments represent measured medial and lateral compartment widths: femoral (dashed), tibial (solid). DXA, dual‐energy X‐ray absorptiometry.

### Clinical outcomes

Binary variables were derived for soft tissue knee injury, meniscus injury, ligament injury and hospital‐diagnosed knee osteoarthritis (HES KOA) using International Classification of Diseases (ICD) codes, which were obtained via linkage to Hospital Episode Statistics (HES; Supporting Information S1: Table 1). Soft tissue knee injury was defined as a composite outcome encompassing both meniscus and ligament injuries to maximize statistical power. Meniscus and ligament injuries were also analysed separately to explore whether associations were driven by a particular injury subtype. Recording of HES data began in April 1997, and outcome data were derived in August 2023, capturing information up to the end of October 2022. Given that knee injury and HES KOA diagnoses could have occurred either before or after the DXA scan, these outcomes were analysed as overall recorded outcomes in the primary analysis, rather than being restricted to post‐scan events. TKR was restricted to events after the scan and based on Office of Population Censuses and Surveys (OPCS) codes. Chronic knee pain (lasting >3 months) was derived as a binary variable, using responses from a questionnaire during the participants' DXA appointment [4].

### Statistical analysis

Descriptive statistics were used to summarize the baseline population characteristics, presented as mean, standard deviation (SD) and range for continuous variables and frequencies for binary variables. All continuous variables were assessed for normality and were normally distributed. Pearson correlation analysis was used to assess the relationship between medial and lateral FTWR. Clustered logistic regression was used to investigate the associations between the exposures of whole FTWR, medial FTWR and lateral FTWR and the outcomes of soft tissue knee injury, meniscus injury, ligament injury, HES KOA, TKR and knee pain, where side‐specific data were unavailable. Each participant provides two FTWR measures to the regression model (one per knee), each paired with the same outcome. By clustering at the participant level, this accounted for the inclusion of bilateral knee measurements, alongside outcomes that were not side‐specific. Odds ratios (ORs) are presented per SD change in FTWR. Models were adjusted for age, sex, height, weight and ethnicity (white/non‐white). Height and weight were modelled separately because bone shape and size are known to be positively correlated with both [20]. Participants' height and weight were measured standing, just prior to the DXA. Ethnicity was self‐reported; due to the low number of non‐white participants, these groups were combined. The primary analysis included both males and females. Additional sex‐interaction terms and sex‐stratified sensitivity analyses were performed. As participants underwent imaging at different times and follow‐up duration varied, Cox proportional hazards modelling was performed as a time‐to‐event sensitivity analysis for TKR, in participants with available data. All analyses were conducted in Stata Version 18.0 (StataCorp).

### Equity, diversity and inclusion

This study included men and women from the UK Biobank, a large, predominantly white (96.5%) population‐based cohort that is known to be affected by healthy‐volunteer selection bias. Therefore, individuals from more deprived backgrounds and some minority ethnic groups are underrepresented [14]. Analyses were adjusted for sex and ethnicity, and sex‐specific analyses were performed. The author team includes an ethnically diverse mix of junior and senior researchers from different disciplines, all based in a single high‐income country.

## RESULTS

### Baseline characteristics

At the start of this study, 63,814 right knee images and 63,486 left knee images were available (Figure 2). Following removal of duplicate records, exclusion of participants with incomplete data linkage and those without bilateral knee measurements, 61,795 participants (123,590 knees) remained (mean age 65.2 years, 52.2% females; mean height 169.8 cm; mean weight 75.3 kg; 96.5% white; Table 1). Mean FTWR was 0.93 (SD 0.02, range 0.81–1.07; Supporting Information S1: Table 2). Mean medial FTWR was higher in males 0.95 (0.07, 0.66–1.31) than females 0.92 (0.08, 0.66–1.41), whereas lateral FTWR was similar between both sexes. Example images of high and low FTWR are provided in Supporting Information S1: Figure 1. FTWR measurements showed a very small mean difference between the right and left knees (−0.001, equivalent to 0.05 SD; *p* < 0.001; Supporting Information S1: Table 3. Medial and lateral compartment measures were strongly negatively correlated (*r* = −0.78, *p* < 0.001). A total of 3010 (4.9%) combined soft tissue knee injuries were identified, with a higher prevalence in males (1738, 5.9%) than in females (1272, 3.9%). Ligament injuries were present in 969 (1.6%) participants, and meniscus injuries in 2238 (3.6%) participants, both more common in males than females. HES KOA was observed in 2389 (3.9%) participants, with a higher prevalence in males (1262, 4.3%) than females (1127, 3.5%). The prevalence of TKR was low and similar between sexes (498, 0.8%). Knee pain was reported by 9175 (14.9%) participants overall, with comparable prevalence between females (4860, 15.1%) and males (4315, 14.6%). The number of events happening pre‐ and post‐scan are reported in Supporting Information S1: Table 4.

**Figure 2:**
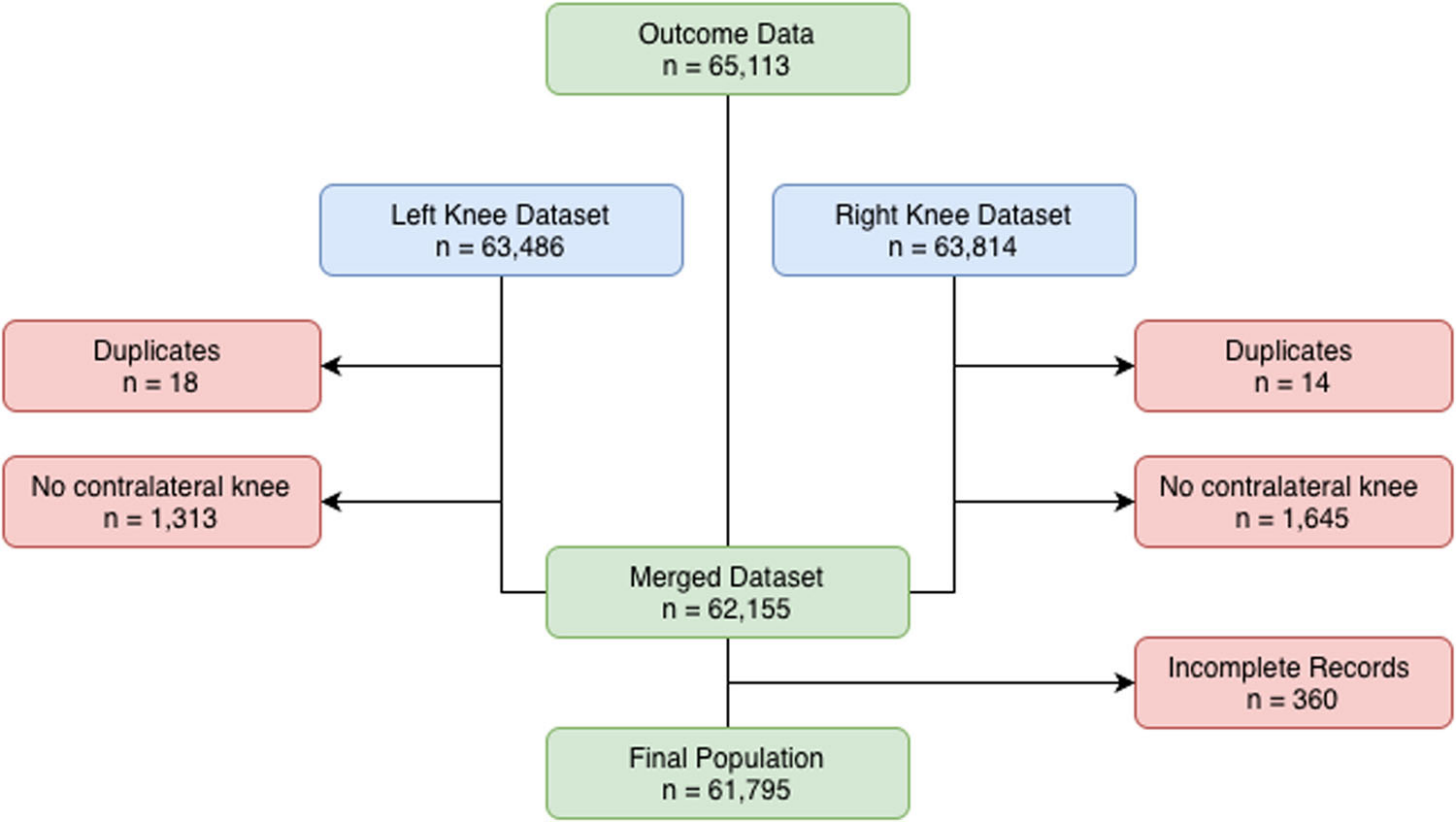
Flow diagram of participant selection showing the number of excluded participants at each stage.

**Table 1:** Descriptive statistics for the study population.

Variable	Combined (mean [SD, range])	Male (mean [SD, range])	Female (mean [SD, range])
Demographics			
Age at imaging (years)	65.2 [7.7, 45–85]	65.9 [7.8, 45–85]	64.5 [7.6, 45–85]
Height (cm)	169.8 [9.4, 124–204]	176.9 [6.6, 148–204]	163.4 [6.4, 124–198]
Weight (kg)	75.3 [15.2, 34–200]	83.0 [13.4, 44–200]	68.2 [13.1, 34–200]
Ethnicity white/non‐white, *n* (%)	59,602 (96.5)	28,480 (96.5)	31,122 (96.4)
Exposures			
Whole FTWR	0.93 [0.02, 0.81–1.07]	0.94 [0.02, 0.82–1.07]	0.93 [0.02, 0.81–1.06]
Medial FTWR	0.94 [0.08, 0.66–1.41]	0.95 [0.07, 0.66–1.31]	0.92 [0.08, 0.66–1.41]
Lateral FTWR	0.94 [0.08, 0.62–1.34]	0.93 [0.07, 0.68–1.27]	0.94 [0.08, 0.62–1.34]

	**Frequency (%)**	**Frequency (%)**	**Frequency (%)**
Knee pain	9175 (14.9)	4315 (14.6)	4860 (15.1)
Knee injury outcomes			
Soft tissue knee injury	3010 (4.9)	1738 (5.9)	1272 (3.9)
Ligament injury	969 (1.6)	487 (1.7)	482 (1.5)
Meniscus injury	2238 (3.6)	1359 (4.6)	879 (2.7)
Knee osteoarthritis outcomes			
Hospital‐diagnosed knee OA	2389 (3.9)	1262 (4.3)	1127 (3.5)
Total knee replacement	498 (0.8)	234 (0.8)	264 (0.8)
Total participants, *n* (%)	61,795 (100)	29,513 (47.8)	32,282 (52.2)

Abbreviations: FTWR, femorotibial width ratio; OA, osteoarthritis; SD, standard deviation.

### Soft tissue knee injury

The adjusted combined‐sex analysis revealed that higher whole‐knee FTWR was associated with increased odds of knee injury across all outcomes (Table 2, Figure 3). For soft tissue knee injury, whole joint FTWR was associated with a greater likelihood of injury (OR 1.09, 95% confidence interval [CI] 1.06–1.13; *p* = 1.4 × 10^−7^). This result was mirrored by the medial compartment, with medial FTWR showing positive associations (OR 1.09, 95% CI 1.06–1.13; *p* = 1.2 × 10^−7^). In contrast, there was little evidence of an association between lateral FTWR and knee injury (OR 0.97, 95% CI 0.94–1.00; *p* = 0.05).

**Table 2:** Adjusted associations between FTWR exposures and soft tissue knee injury outcomes.

	Combined knee injury		Meniscus injury		Ligament injury	
Exposure	OR [95% CI]	*p* value	OR [95% CI]	*p* value	OR [95% CI]	*p* value
Whole FTWR	1.09 [1.06–1.13]	1.4 × 10^−7^	1.10 [1.05–1.14]	4.9 × 10^−6^	1.07 [1.01–1.13]	0.02
Medial FTWR	1.09 [1.06–1.13]	1.2 × 10^−7^	1.11 [1.07–1.16]	3.3 × 10^−8^	1.04 [0.98–1.10]	0.21
Lateral FTWR	0.97 [0.94–1.00]	0.05	0.95 [0.92–0.99]	0.01	1.01 [0.95–1.06]	0.81

*Note*: Results presented as OR per SD change in FTWR.

Abbreviations: CI, confidence interval; FTWR, femorotibial width ratio; OR, odds ratio.

**Figure 3:**
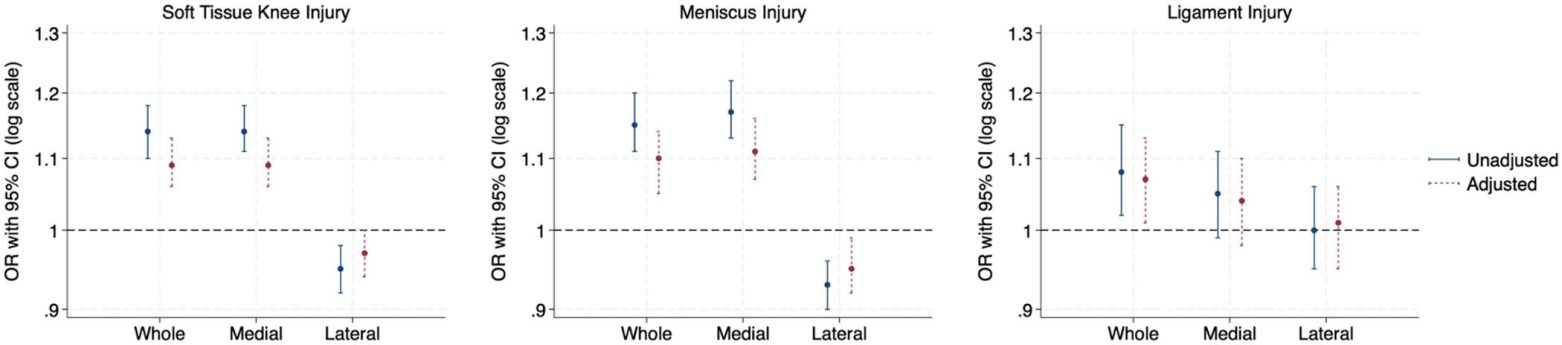
Associations between whole, medial and lateral femorotibial width ratio (FTWR) and knee soft tissue injury in a sex combined analysis. Clustered logistic regression was used. Results are presented as odds ratios (ORs) per standard deviation change in FTWR. Models were adjusted for age, sex, height, weight and ethnicity (white/non‐white).

When ligament and meniscus injuries were examined separately, greater whole FTWR was associated with an increased likelihood of ligament (OR 1.07, 95% CI 1.01–1.13; *p* = 0.02) and meniscus injury (OR 1.10, 95% CI 1.05–1.14; *p* = 4.9 × 10^−6^). Medial FTWR showed a strong association with meniscus injury (OR 1.11, 95% CI 1.07–1.16; *p* = 3.3 × 10^−8^), whereas there was little evidence of an association with ligament injury (OR 1.04, 95% CI 0.98–1.10; *p* = 0.21). Lateral FTWR showed little evidence of being associated with meniscus injury (OR 0.95, 95% CI 0.92–0.99; *p* = 0.01) and was not associated with ligament injury (OR 1.01, 95% CI 0.95–1.06; *p* = 0.81). There was no evidence that the associations between whole FTWR and combined soft tissue knee injury (*p* interaction = 0.50) or meniscus injury (*p* interaction = 0.58) differed by sex. For ligament injury, there was some evidence of a stronger association in males (OR 1.14, 95% CI 1.05–1.24) than in females (OR 1.02, 95% CI 0.94–1.10; *p* interaction = 0.04; Supporting Information S1: Table 5).

### Knee osteoarthritis

In the adjusted combined‐sex analysis, greater whole FTWR showed strong evidence of a positive association with KOA outcomes (Table 3, Figure 4): HES KOA (OR 1.16, 95% CI 1.12–1.21; *p* = 9.2 × 10^−15^), TKR (OR 1.27, 95% CI 1.17–1.39; *p* = 4.9 × 10^−8^) and chronic knee pain (OR 1.08, 95% CI 1.05–1.10; *p* = 1.8 × 10^−12^). These associations were largely attributable to the medial compartment. Greater medial FTWR was associated with an increased likelihood of HES KOA (OR 1.11, 95% CI 1.07–1.15; *p* = 3.3 × 10^−8^), TKR (OR 1.22, 95% CI 1.13–1.32; *p* = 2.7 × 10^−7^) and knee pain (OR 1.04, 95% CI 1.01–1.06; *p* = 7.6 × 10^−4^). In contrast, there was no evidence that lateral FTWR was associated with osteoarthritis outcomes (HES KOA [OR 0.99, 95% CI 0.96–1.03; *p* = 0.78], TKR [OR 0.95, 95% CI 0.88–1.03; *p* = 0.22] or knee pain [OR 1.01, 95% CI 0.99–1.03; *p* = 0.16]).

**Table 3:** Adjusted associations between FTWR exposures and knee osteoarthritis outcomes.

	HES KOA		TKR		Knee pain	
Exposure	OR [95% CI]	*p* value	OR [95% CI]	*p* value	OR [95% CI]	*p* value
Whole FTWR	1.16 [1.12–1.21]	9.2 × 10^−15^	1.27 [1.17–1.39]	4.9 × 10^−8^	1.08 [1.05–1.10]	1.8 × 10^−12^
Medial FTWR	1.11 [1.07–1.15]	3.3 × 10^−8^	1.22 [1.13–1.32]	2.7 × 10^−7^	1.04 [1.01–1.06]	7.6 × 10^−4^
Lateral FTWR	0.99 [0.96–1.03]	0.78	0.95 [0.88–1.03]	0.22	1.01 [0.99–1.03]	0.16

*Note*: Results are presented as OR per standard deviation change in FTWR.

Abbreviations: CI, confidence interval; FTWR, femorotibial width ratio; HES KOA, hospital‐diagnosed knee osteoarthritis; OR, odds ratio; TKR, total knee replacement.

**Figure 4:**
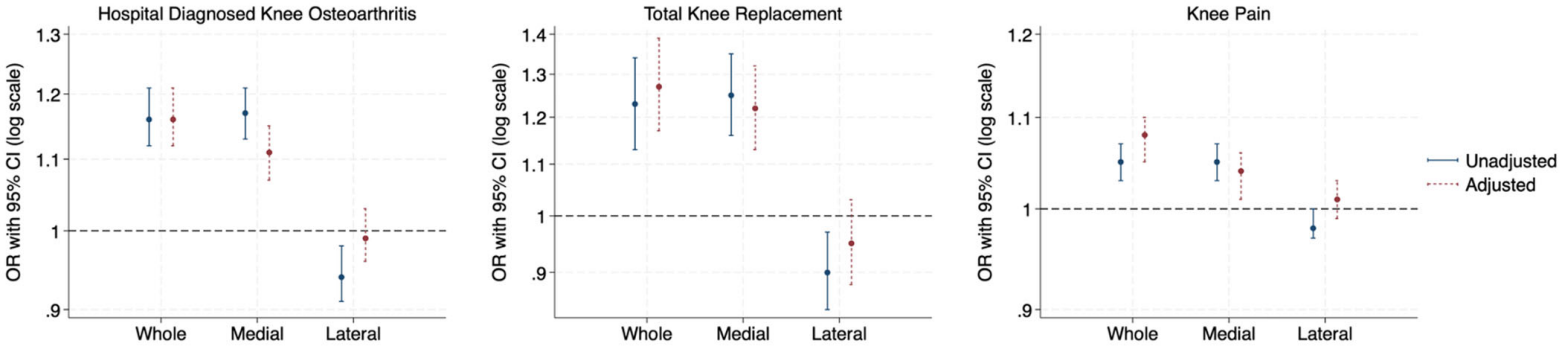
Associations between whole, medial and lateral femorotibial width ratio (FTWR) and knee osteoarthritis in a sex combined analysis. Clustered logistic regression was used. Results are presented as odds ratios (ORs) per standard deviation change in FTWR. Models were adjusted for age, sex, height, weight and ethnicity (white/non‐white). CI, confidence interval.

Sex‐stratified analysis showed broadly similar patterns in males and females, with consistent associations for whole FTWR, and similar but weaker associations for medial FTWR (Supporting Information S1: Table 6). There was no evidence of effect modification by sex for the associations between whole FTWR and HES KOA (*p* interaction = 0.97), TKR (*p* interaction = 0.63) or knee pain (*p* interaction = 0.88). For TKR, the time‐to‐event analysis showed similar associations with FTWR as those observed in the primary analysis (Supporting Information S1: Table 7).

## DISCUSSION

In this large population‐based study of 61,795 UK Biobank participants, FTWR was consistently associated with both soft tissue knee injury and KOA. Greater whole FTWR was associated with increased likelihood of soft tissue, meniscus and ligament injury. Stronger associations were observed for osteoarthritis outcomes, including HES KOA, TKR and chronic knee pain. These associations were primarily driven by the medial compartment, as lateral FTWR showed largely null or weak inverse associations.

This is the first population‐scale study to examine the associations between femorotibial width discrepancy and both knee injury and osteoarthritis. Previous work investigating structural risk factors for knee injury has focused on specific anatomical features. However, these studies tended to be relatively small and frequently limited to athletic populations [17, 18, 33, 40, 41, 43]. Morphological risk factors for knee injury have centred on features that may predispose to instability or altered kinematics. For example, increased posterior tibial slope has been linked to greater anterior tibial translation under load, thereby increasing strain on the ACL [18]. Similarly, a pyramidal‐shaped intercondylar notch, compared with a smooth or dome shape, has been associated with better knee stability, due to increased bone congruity [26]. While these anatomical features have been implicated in knee injury risk, many require complex imaging measurements, making them difficult to quantify consistently in large population‐based studies.

FTWR differs conceptually from these measures and represents a simple measure of the relative width of the femur and tibia across the knee joint. Rather than focusing on discrete anatomical landmarks, it reflects the proportional relationship between the femoral and tibial articular surfaces. Recent work examining limb proportions, comparing the length of the tibia and femur, has shown that a lower tibia:femur ratio (TFR) is associated with ACL injury [32]. The authors proposed that this is due to changes in knee loading patterns and altered centre of mass. Similarly, a relatively wider femur compared to the tibia could plausibly reduce joint congruence and alter contact mechanics across the knee joint. These changes may influence not only increased ligament strain, but also meniscal loading and stress on articular cartilage; however, further research is needed to confirm the exact mechanisms behind this. FTWR has the advantage of being anatomically measurable and compartment‐specific, in contrast to statistical shape modelling approaches [3, 36], which summarize complex aspects of joint morphology. That said, FTWR and other simple geometric measures likely capture only a portion of the underlying joint morphology. In this context, more complex modelling approaches may provide more nuanced insight into joint geometry and biomechanical risk. Further studies are justified to identify the optimal modelling technique for clinical practice.

The associations between FTWR and injury outcomes were modest in their magnitude, which is consistent with the multifactorial nature of knee injury [10, 45]. Nevertheless, the presence of associations in a large, population‐based cohort suggests that femorotibial width discrepancy represents a measurable component of structural risk. In addition to injury outcomes, higher FTWR was associated with increased odds of multiple osteoarthritis‐related measures, including HES KOA, TKR and knee pain. These associations were stronger than those observed for soft tissue injury and were driven largely by the medial compartment, while lateral FTWR showed little or weak negative associations with knee injury and osteoarthritis. The strong negative correlation observed between medial and lateral FTWR may explain this association. This pattern is notable given the predominance of medial compartment loading during gait [25] and the higher prevalence of medial compartment osteoarthritis [42, 49]. It is also plausible that changes in medial FTWR have greater biomechanical consequences.

One potential explanation for this is that increased FTWR influences long‐term load distribution across the joint. Three‐dimensional models, created from computed tomography scans of patients with medial compartment KOA, have shown more posterior, lateral and proximal translation of the tibia relative to the femur, when compared with the contralateral side [24]. These changes also aligned with more medial shifts in contact patterns of the tibial plateau. Therefore, a larger relative width of the femur compared to the tibia could alter contact area, over time leading to increased degeneration of articular cartilage. FTWR could reflect underlying developmental differences in knee morphology that may be associated with injury and degenerative change. UK Biobank has paired multiomic data for each participant, which means future genetic studies could delve into the biological aetiology of altered FTWR [12, 13].

The observation that FTWR is associated with both injury and osteoarthritis outcomes aligns with the well‐established link between knee injury and subsequent development of osteoarthritis [16, 34, 48]. It may be that higher FTWR increases the risk of knee injury, which then contributes to accelerated joint degeneration [8]. Alternatively, FTWR may directly increase the risk of osteoarthritis due to changes in joint biomechanics, independent of injury. Further longitudinal studies are needed to clarify the temporality of the associations reported in this study to better understand underlying aetiological mechanisms. The observational and mostly cross‐sectional nature of this study cannot rule out residual confounding and reverse causation, meaning causal inferences should not be made. The repeat imaging of UK Biobank participants, currently underway, will allow for this question to be answered in time.

### Clinical implications

FTWR is derived from standardised DXA images using automated methods, making it highly scalable within large imaging datasets. Although paired radiographic images were not available to compare X‐ray‐derived FTWR with DXA‐derived FTWR in this cohort, with further study these methods may potentially be transferable to traditional knee radiographs that are more widely available in clinical and research contexts [51]. These findings suggest that a simple morphological measure such as FTWR is associated with knee injury and osteoarthritis and could provide a basis for early risk stratification.

Whole FTWR was associated with 27% increased odds of TKR per SD increase in score. Meaning, those with an FTWR greater than 2 SD translates to approximately 2.6‐fold higher odds of TKR when compared to individuals with an FTWR 2 SD below the mean, suggesting that this association may be clinically meaningful across the exposure distribution. However, the associations with soft tissue injury are more modest, with a 41% increased odds across the same distribution, yet this could still be an important difference when considering individualized injury prevention in the future.

DXA is already well established in elite sport for assessing body composition, providing an opportunity to study FTWR in populations at high risk of knee injury [1, 23, 31, 44]. However, the study cohort consisted of middle‐aged and older adults from a general population. Prospective studies in younger and more active populations are warranted to determine whether FTWR can be used to support early identification of individuals at increased risk of knee injury or degenerative changes and implement targeted early preventative strategies [46].

### Limitations

There are several limitations to consider in this study. Injury and osteoarthritis outcomes were identified using HES and OPCS codes, which are not side‐specific and may underestimate less severe cases managed in primary care. Despite the relatively coarse nature of routinely collected healthcare data, the consistent direction of associations across these different phenotypes provides some reassurance that the observed relationship between FTWR and KOA is not dependent on a single coded phenotype. In addition, any remaining outcome misclassification would generally be expected to bias associations towards the null. The HES data available for this analysis began in April 1997; therefore, injuries and procedures occurring before this may not be captured. This is particularly relevant in the UK Biobank population, in which historical knee injuries may have occurred several decades before imaging. The temporal relationship between FTWR and HES injury and KOA outcomes remains an important limitation. These diagnoses may have occurred before or after DXA imaging, and HES coding dates may not correspond to the exact onset of disease, particularly for KOA, which is a chronic and progressive condition. Although adjustments for key demographic factors were performed as sensitivity analyses, the observational study design and potential for residual confounding mean that causal inference remains limited.

Knee positioning was standardised during image acquisition; however, some residual variation in knee rotation may have remained. As DXA provides a two‐dimensional representation of three‐dimensional anatomy, these differences may influence the measured femoral and tibial widths and therefore FTWR. In addition, the mechanical axis of the femur and tibia may not be perfectly collinear in individual knees, and this two‐dimensional approach does not fully account for variations in alignment. However, this pre‐defined approach was applied consistently across all images. Further work is justified to confirm these associations using three‐dimensional imaging.

Landmark placement on rounded contours (e.g., point 16) can be difficult, and point‐specific validation data were unavailable. However, measurement error would likely bias results towards the null rather than induce false positive associations. The placement of landmark points may not fully account for condylar remodelling and changes to subchondral bone that occur with established KOA; as a result, the observed association between higher FTWR and KOA outcomes may partly reflect reverse causation. Further replication of these associations in external cohorts is warranted, and longitudinal studies with pre‐disease baseline imaging or the use of genetic instruments in Mendelian randomization in particular, could help further clarify these associations [12, 13]. Participants with previous fractures were not specifically excluded, and post‐traumatic deformities may affect landmark placement in some individuals. That said, all images were manually reviewed, and such deformities were not obviously seen. Finally, UK Biobank is predominantly white (96.5%) and has lower rates of all‐cause mortality than the general population [14]. Combining non‐white participants into a single group prevents examining potential differences in FTWR across specific ethnic groups. These factors may limit the generalizability of the findings to more ethnically diverse populations.

## CONCLUSION

In conclusion, this study shows that greater FTWR was associated with an increased likelihood of soft tissue knee injury and osteoarthritis, with the strongest and most consistent associations observed in the medial compartment. Future work should focus on whether FTWR can serve as a scalable imaging marker for risk stratification and targeted prevention.

## AUTHOR CONTRIBUTIONS

Bilal Rayes, Mijin Jung and Benjamin G. Faber led the analysis and drafted the manuscript. Benjamin G. Faber and Bilal Rayes developed the Python pipeline for image processing. Benjamin G. Faber and Martyn Snow conceived the study and provided overall supervision. Mijin Jung contributed to statistical analysis and interpretation of the data. Bilal Rayes, Fiona Saunders, Raja Ebsim, Richard M. Aspden, Jenny Gregory, Timothy Cootes, Claudia Lindner, Simon G. F. Abram and Jonathan H. Tobias contributed to study design, interpretation of the findings and critically revised the manuscript for important intellectual content. All authors reviewed and approved the final manuscript. Bilal Rayes is the guarantor and accepts full responsibility for the integrity of the work.

## CONFLICT OF INTEREST STATEMENT

The authors declare no conflicts of interest.

## ETHICS STATEMENT

The UK Biobank was given ethical approval by the National Information Governance Board for Health and Social Care and the North‐West Multi‐Centre Research Ethics Committee (11/NW/0382). All participants provided their informed consent for their data to be used in the study and followed up through linkage with healthcare‐related records.

## Supporting information

Supporting File 1.

## Data Availability

All UK Biobank data will be available via their data showcase shortly after publication.
