# Supplementary material for: Higher femorotibial width ratio is associated with knee injury and osteoarthritis: A UK Biobank study of 61,795 participants
Source: J Exp Orthop. 2026 Nov 13;13(4):e70986. doi: 10.1002/jeo2.70986 (PMC13766272; doi:10.1002/jeo2.70986)
Supplement: Supplementary file 1 [file JEO2-13-e70986-s001.docx]

**Supplementary Figure 1.** A. Left knee DXA scan showing an individual with a femorotibial width ratio + 3.5 standard deviations from the mean. B. Left knee DXA scan showing an individual with a femorotibial width ratio -3.3 standard deviations from the mean. Reproduced by kind permission of UK Biobank ©.

**Supplementary Table 1.** ICD and OPCS-4 codes used to define knee injury outcomes. Soft tissue knee injury was defined as a composite outcome including both meniscus and ligament injuries identified using the ICD and OPCS-4 codes listed below.

| **Coding system** | **Code** | **Description** | **Outcome classification** |
| --- | --- | --- | --- |
| **ICD-9** | | | |
| ICD-9 | 71536 | Osteoarthrosis, localized, lower leg | Knee osteoarthritis |
| ICD-9 | 71516 | Localised, primary osteoarthrosis and allied disorders, lower leg | Knee osteoarthritis |
| **ICD-10** | | | |
| ICD-10 | S80 | Contusion of knee | Ligament injury |
| ICD-10 | S83 | Dislocation, sprain and strain of joints and ligaments of knee | Ligament injury |
| ICD-10 | S832 | Tear of meniscus | Meniscus injury |
| ICD-10 | S833 | Tear of articular cartilage of knee | Meniscus injury |
| ICD-10 | S834 | Sprain/strain of collateral ligament | Ligament injury |
| ICD-10 | S835 | Sprain/strain of cruciate ligament | Ligament injury |
| ICD-10 | S836 | Sprain/strain of other knee structures | Ligament injury |
| ICD-10 | M232 | Derangement of meniscus due to old tear | Meniscus injury |
| ICD-10 | M233 | Other meniscus derangements | Meniscus injury |
| ICD-10 | M236 | Spontaneous disruption of knee ligaments | Ligament injury |
| ICD-10 | M17 | Gonarthrosis (arthrosis of knee). | Knee osteoarthritis |
| ICD-10 | M170 | Primary gonarthrosis, bilateral. | Knee osteoarthritis |
| ICD-10 | M171 | Other primary gonarthrosis | Knee osteoarthritis |
| ICD-10 | M179 | Gonarthrosis, unspecified | Knee osteoarthritis |
| ICD-10 | M1906 | Primary arthrosis of other joints, lower leg | Knee osteoarthritis |
| ICD-10 | M1996 | Arthrosis, unspecified, lower leg | Knee osteoarthritis |
| **OPCS-4 (Meniscus)** | | | |
| OPCS-4 | W70 | Open operations on meniscus | Meniscus injury |
| OPCS-4 | W701 | Open total excision of meniscus | Meniscus injury |
| OPCS-4 | W702 | Open excision of meniscus NEC | Meniscus injury |
| OPCS-4 | W703 | Open repair of meniscus | Meniscus injury |
| OPCS-4 | W704 | Open total replacement of meniscus | Meniscus injury |
| OPCS-4 | W705 | Open partial replacement of meniscus | Meniscus injury |
| OPCS-4 | W708 | Other specified | Meniscus injury |
| OPCS-4 | W709 | Unspecified | Meniscus injury |
| OPCS-4 | W82 | Endoscopic operations on meniscus | Meniscus injury |
| OPCS-4 | W821 | Endoscopic total excision meniscus | Meniscus injury |
| OPCS-4 | W822 | Endoscopic resection of meniscus | Meniscus injury |
| OPCS-4 | W823 | Endoscopic repair of meniscus | Meniscus injury |
| OPCS-4 | W825 | Endoscopic partial replacement of meniscus | Meniscus injury |
| OPCS-4 | W828 | Other specified | Meniscus injury |
| OPCS-4 | W829 | Unspecified | Meniscus injury |
| **OPCS-4 (Ligament)** | | | |
| OPCS-4 | W72 | Prosthetic replacement of ligament | Ligament injury |
| OPCS-4 | W721 | Primary prosthetic replacement of multiple ligaments | Ligament injury |
| OPCS-4 | W722 | Prosthetic replacement of multiple ligaments NEC | Ligament injury |
| OPCS-4 | W723 | Primary prosthetic replacement of intra-articular ligament | Ligament injury |
| OPCS-4 | W724 | Prosthetic replacement of intra-articular ligament NEC | Ligament injury |
| OPCS-4 | W725 | Primary prosthetic replacement of extra-articular ligament | Ligament injury |
| OPCS-4 | W726 | Prosthetic replacement of extra-articular ligament NEC | Ligament injury |
| OPCS-4 | W728 | Other specified | Ligament injury |
| OPCS-4 | W729 | Unspecified | Ligament injury |
| OPCS-4 | W73 | Prosthetic reinforcement of ligament | Ligament injury |
| OPCS-4 | W731 | Primary extra-articular prosthetic augmentation of intra-articular ligament NEC | Ligament injury |
| OPCS-4 | W732 | Extra-articular prosthetic augmentation of intra-articular ligament NEC | Ligament injury |
| OPCS-4 | W733 | Primary prosthetic reinforcement of intra-articular ligament NEC | Ligament injury |
| OPCS-4 | W734 | Prosthetic reinforcement of intra-articular ligament NEC | Ligament injury |
| OPCS-4 | W735 | Primary prosthetic reinforcement of extra-articular ligament NEC | Ligament injury |
| OPCS-4 | W736 | Prosthetic reinforcement of extra-articular ligament NEC | Ligament injury |
| OPCS-4 | W738 | Other specified | Ligament injury |
| OPCS-4 | W739 | Unspecified | Ligament injury |
| OPCS-4 | W74 | Reconstruction of ligament | Ligament injury |
| OPCS-4 | W741 | Reconstruction of multiple ligaments NEC | Ligament injury |
| OPCS-4 | W742 | Reconstruction of intra-articular ligament NEC | Ligament injury |
| OPCS-4 | W743 | Reconstruction of extra-articular ligament NEC | Ligament injury |
| OPCS-4 | W748 | Other specified | Ligament injury |
| OPCS-4 | W749 | Unspecified | Ligament injury |
| OPCS-4 | W75 | Open repair of ligament | Ligament injury |
| OPCS-4 | W751 | Open repair of multiple ligaments NEC | Ligament injury |
| OPCS-4 | W752 | Open repair of intra-articular ligament NEC | Ligament injury |
| OPCS-4 | W753 | Open repair of extra-articular ligament NEC | Ligament injury |
| OPCS-4 | W758 | Other specified | Ligament injury |
| OPCS-4 | W759 | Unspecified | Ligament injury |
| OPCS-4 | W77 | Stabilising operations on joint | Ligament injury |
| OPCS-4 | W771 | Repair of capsule of joint for stabilisation | Ligament injury |
| OPCS-4 | W772 | Transposition of muscle for stabilisation | Ligament injury |
| OPCS-4 | W776 | Annular ligament reconstruction | Ligament injury |
| OPCS-4 | W777 | Transposition of ligament for stabilisation | Ligament injury |
| OPCS-4 | W778 | Other specified | Ligament injury |
| OPCS-4 | W779 | Unspecified | Ligament injury |
| OPCS-4 | W84 | Endoscopic operations on joint structures | Ligament injury |
| OPCS-4 | W841 | Endoscopic repair of intra-articular ligament | Ligament injury |
| OPCS-4 | W842 | Endoscopic reattachment of intra-articular ligament | Ligament injury |
| OPCS-4 | W844 | Endoscopic decompression of joint | Ligament injury |
| OPCS-4 | W848 | Other specified | Ligament injury |
| OPCS-4 | W849 | Unspecified | Ligament injury |
| **OPCS-4 Knee (Osteoarthritis)** | | | |
| OPCS-4 | W401 | Total prosthetic replacement of knee joint using cement | Knee osteoarthritis |
| OPCS-4 | W411 | Total prosthetic replacement of knee joint not using cement | Knee osteoarthritis |
| OPCS-4 | W421 | Other total prosthetic replacement of knee joint | Knee osteoarthritis |

ICD-9: International Classification of Diseases, 9th Revision; ICD-10: International Classification of Diseases, 10th Revision; OPCS-4: Office of Population Censuses and Surveys Classification of Surgical Operations and Procedures, version 4; NEC: Not Elsewhere Classified.

**Supplementary Table 2.** Mean (standard deviation) femorotibial width ratio measures for right and left knees with and without knee injury, and with and without osteoarthritis.

| **Measure** | **No OA**  **(n = 51,366)** | **OA**  **(n=10,429)** | **No Injury**  **(n=58,785)** | **Injury**  **(n=3,010)** |
| --- | --- | --- | --- | --- |
| Whole FTWR (R) | 0.935 (0.02) | 0.937 (0.03) | 0.935 (0.02) | 0.939 (0.03) |
| Whole FTWR (L) | 0.936 (0.02) | 0.937 (0.03) | 0.936 (0.03) | 0.939 (0.03) |
| Medial FTWR (R) | 0.923 (0.08) | 0.929 (0.08) | 0.924 (0.08) | 0.935 (0.08) |
| Medial FTWR (L) | 0.946 (0.08) | 0.949 (0.08) | 0.946 (0.08) | 0.956 (0.08) |
| Lateral FTWR (R) | 0.949 (0.07) | 0.947 (0.08) | 0.949 (0.08) | 0.945 (0.08) |
| Lateral FTWR (L) | 0.929 (0.07) | 0.928 (0.08) | 0.929 (0.08) | 0.925 (0.07) |

OA: osteoarthritis; FTWR: femorotibial width ratio

**Supplementary Table 3.** Femorotibial width ratio measurements compared between right and left knees. Mean differences represent the difference between right and left knees (right minus left), assessed using paired t-tests.

.

|  | **Right Knee** (n = 61,795) | **Left Knee**  (n = 61,795) | **Mean Difference** | **P-value** |
| --- | --- | --- | --- | --- |
| **Exposures (mean [range])** |  |  |  |  |
| Whole FTWR | 0.93 [0.83-1.07] | 0.93 [0.81-1.06] | −0.0010 | <0.001 |
| Medial FTWR | 0.92 [0.66-1.31] | 0.95 [0.66-1.41] | −0.0221 | <0.001 |
| Lateral FTWR | 0.95 [0.66-1.34] | 0.93 [0.62-1.29] | +0.0202 | <0.001 |

FTWR: femorotibial width ratio

**Supplementary Table 4.** Pre-and post-scan incidence for knee osteoarthritis and soft tissue knee injury outcomes.

| **Outcome** | **Total Events** | **Pre-scan Events** | **Post-scan Events** |
| --- | --- | --- | --- |
| HES-KOA | 2389 | 1586 | 803 |
| TKR | 498 | 0 | 498 |
| Knee Pain | 9175 | * | * |
| Combined Knee Injury | 3010 | 2757 | 253 |
| Meniscus Injury | 2238 | 2086 | 152 |
| Ligament Injury | 969 | 847 | 122 |

HES KOA: hospital-diagnosed knee osteoarthritis; TKR: total knee replacement; * assessed contemporaneously (on DXA visit), pre/post-split does not apply.

**Supplementary Table 5.** Sex-stratified associations between femorotibial width ratio exposures and soft tissue knee injury outcomes.

|  | **Combined knee injury** | | **Meniscus Injury** | | **Ligament Injury** | |
| --- | --- | --- | --- | --- | --- | --- |
| **Exposure** | **OR [95% CI]** | ***p*-value** | **OR [95% CI]** | ***p*-value** | **OR [95% CI]** | ***p*-value** |
| **Males** |  |  |  |  |  |  |
| Whole FTWR | 1.10 [1.05-1.16] | 3.4 × 10^-5^ | 1.08 [1.03-1.14] | 3.5 × 10^-3^ | 1.14 [1.05-1.24] | 2.4 × 10^-3^ |
| Medial FTWR | 1.11 [1.06-1.16] | 4.4 × 10^-6^ | 1.11 [1.06-1.17] | 2.4 × 10^-5^ | 1.09 [1.03-1.15] | 0.10 |
| Lateral FTWR | 0.96 [0.92-1.00] | 0.043 | 0.94 [0.90-0.99] | 0.013 | 0.99 [0.95-1.04] | 0.75 |
| **Females** |  |  |  |  |  |  |
| Whole FTWR | 1.09 [1.03-1.14] | 9.6 × 10^-4^ | 1.12 [1.05-1.19] | 2.8 × 10^-4^ | 1.02 [0.94-1.10] | 0.62 |
| Medial FTWR | 1.08 [1.02-1.13] | 4.0 × 10^-3^ | 1.12 [1.05-1.19] | 3.2 × 10^-4^ | 1.01 [0.93-1.09] | 0.88 |
| Lateral FTWR | 0.98 [0.94-1.03] | 0.046 | 0.97 [0.95-1.06] | 0.26 | 1.00 [0.93-1.08] | 0.96 |

FTWR: femorotibial width ratio; OR: Odds ratio; CI: confidence interval.

**Supplementary Table 6.** Sex-stratified associations between femorotibial width ratio exposures and knee osteoarthritis outcomes.

|  | **HES KOA** | | **TKR** | | **Knee Pain** | |
| --- | --- | --- | --- | --- | --- | --- |
| **Exposure** | **OR [95% CI]** | ***p*-value** | **OR [95% CI]** | ***p*-value** | **OR [95% CI]** | ***p*-value** |
| **Males** |  |  |  |  |  |  |
| Whole FTWR | 1.16 [1.10-1.23] | 8.0 × 10^-8^ | 1.30 [1.14-1.48] | 5.8 × 10^-5^ | 1.07 [1.04-1.10] | 1.8 × 10^-5^ |
| Medial FTWR | 1.12 [1.06-1.18] | 4.6 × 10^-5^ | 1.22 [1.09-1.36] | 3.5 × 10^-4^ | 1.04 [1.01-1.07] | 0.012 |
| Lateral FTWR | 0.98 [0.94-1.04] | 0.55 | 0.96 [0.86-1.08] | 0.49 | 1.00 [0.97-1.03] | 0.78 |
| **Females** |  |  |  |  |  |  |
| Whole FTWR | 1.17 [1.11-1.23] | 2.5 × 10^-8^ | 1.25 [1.11-1.40] | 2.0 × 10^-4^ | 1.08 [1.05-1.11] | 3.3 × 10^-8^ |
| Medial FTWR | 1.11 [1.05-1.17] | 1.7 × 10^-4^ | 1.22 [1.10-1.36] | 1.8 × 10^-4^ | 1.03 [1.01-1.06] | 0.020 |
| Lateral FTWR | 1.00 [0.95-1.06] | 0.88 | 0.95 [0.86-1.05] | 0.29 | 1.02 [0.99-1.05] | 0.13 |

HES KOA: hospital-diagnosed knee osteoarthritis; TKR: total knee replacement; FTWR: femorotibial width ratio; OR: Odds ratio; CI: confidence interval.

**Supplementary Table 7.** Associations between femorotibial width ratio exposures and total knee replacement using a time to event clustered Cox proportional hazard modelling. Hazard ratios (HR) are presented per standard deviation change in FTWR. Mean follow up 3.95 years, range 0.00-7.99 years.

|  | **TKR (unadjusted)** | | **TKR (adjusted)** | |
| --- | --- | --- | --- | --- |
| **Exposure** | **HR [95% CI]** | ***p*-value** | **HR [95% CI]** | ***p*-value** |
| Whole FTWR | 1.08 [1.00-1.18] | 5.0 × 10^-2^ | 1.11 [1.03-1.21] | 1.0 × 10^-2^ |
| Medial FTWR | 1.19 [1.10-1.28] | 1.0 × 10^-5^ | 1.23 [1.05-1.23] | 1.5 × 10^-3^ |
| Lateral FTWR | 0.88 [0.81-0.95] | 9.8 × 10^-4^ | 0.95 [0.88-1.02] | 0.16 |

Adjusted for age, sex, height, weight and ethnicity (white/non-white). Linkage to healthcare records ended before some participants were scanned. Therefore, these sensitivity analyses were conducted in a subset of 50,730 individuals where data linkage occurred after the date of scan. TKR: total knee replacement; HR: Hazard ratio; CI: confidence interval.
